# Accountability for carbon emissions and health equity 

**DOI:** 10.2471/BLT.22.289452

**Published:** 2023-02-01

**Authors:** Sarah Whitmee, Blanca Anton, Andy Haines

**Affiliations:** a Centre on Climate Change and Planetary Health, London School of Hygiene and Tropical Medicine, London WCIE 7HT, England.

A new database of anthropogenic greenhouse gas emissions can improve the accountability of climate change mitigation actions, while promoting human health and equity, supporting a just transition to a net zero emission future and reducing the risks of climate change. Climate TRACE uses data from 300 satellites and more than 11 100 air-, land- and sea-based sensors, together with other commercial and public sources to map sources of greenhouse gas emissions.^1^ Climate TRACE includes estimated emissions of carbon dioxide, methane and nitrous oxide, and allows the comparison of their global warming potential over the next 20 and 100 years. The aim of Climate TRACE is to focus on major sources of emissions such as those from power plants, oil fields and road transportation, rather than from the daily activities of individuals. According to Climate TRACE estimates, power plants are responsible for over half of the emissions and constitute three fifths of the top 500 emission sources. Oil and gas fields, together with their associated production, processing and transportation sites, comprise 26 of the 50 largest sources of emissions worldwide.^1^

Current greenhouse gas emissions are too high to achieve the Paris Agreement goal of limiting global average temperature increase to 1.5 °C.^2^ The creation of such a public repository can help to increase the accountability of the world’s biggest polluters. Under the United Nations Framework Convention on Climate Change, countries are responsible for self-reporting their greenhouse gas emissions, which often results in underreporting or outdated inventories. For example, estimated emissions from oil and gas production and refining among top countries that submit inventories regularly are now thought to be more than double the previously reported estimates.^3^

People who contribute least to global emissions are already disproportionately affected by climate change, including through a range of direct and indirect impacts on health.^4^ These impacts include the effects of increasing exposure to extreme heat, wildfires, floods and droughts, increased exposure to a range of infectious diseases, food insecurity, undernutrition, poverty and population displacement.^5^ Approximately 771 million people were responsible for nearly half of greenhouse gas emissions in 2019, while half of the world population emitted only about one tenth of total of global emissions.^6^ Rigorous emissions data may feed into future negotiations around payment for loss and damage – that is, the harm caused by anthropogenic climate change in low- and middle-income countries – as agreed in principle at the Conference of Parties 27. Attributing deaths to human-induced climate change as distinct from natural climate variability^7^ is increasingly possible, giving credibility to claims for payments in compensation for losses from the effects of climate change.

Linking the impacts of these mapped emissions to health exposures and outcomes could also stimulate action leading to near-term health benefits from reduced emissions. The burning of fossil fuels is a major contributor to ambient air pollution and is responsible for millions of premature deaths annually.^8^^,^^9^ Phasing out fossil fuels will both reduce the risks of climate change and deaths from air pollution.

However, Climate TRACE data currently have limitations. For example, data only cover production emissions whereas ideally, the estimated emissions related to goods and services consumed in a particular country should also be included. Addressing the ultimate drivers of emissions, including overconsumption in high-income countries, is essential to achieve the change needed to reach net zero. Well-designed multisectoral actions can accelerate progress towards net zero and sustain and improve health, but current actions are inadequate. Rapid greenhouse gas mitigation provides an opportunity for global health as it can reduce health risks posed by climate change while delivering benefits to human health and development. Alongside the benefits from cleaner air, benefits to health can also be achieved through increased physical activity from active travel and healthier, more sustainable diets.^10^^,^^11^

To realize these benefits, existing barriers and challenges to action must be addressed, including the use of standard approaches to measuring and reporting on the health effects of climate mitigation actions.^12^ Evidence from different studies from the same sector is currently difficult to compare. As a result, assessing the effectiveness of mitigation actions is difficult. Climate TRACE is an important step in supporting the accountability and reporting of greenhouse gas emissions and the scope should be expanded to include metrics reflecting human health and equity. Reframing climate change as a health issue and emphasizing the potential of climate mitigation actions to improve health could accelerate the implementation of ambitious actions to achieve the Paris Climate Agreement goals.
